# Risk Factors of Recurrence after Stent(s)-Assisted Coiling of Intracranial Vertebrobasilar Dissecting Aneurysms: A Multicenter Study

**DOI:** 10.3389/fneur.2017.00482

**Published:** 2017-09-14

**Authors:** Kun Wang, Zhongbin Tian, Junfan Chen, Jian Liu, Yang Wang, Hongqi Zhang, Jun Wang, Yisen Zhang, Xinjian Yang

**Affiliations:** ^1^Department of Interventional Neuroradiology, Beijing Neurosurgical Institute, Beijing Tiantan Hospital, Capital Medical University, Beijing, China; ^2^Department of Neurosurgery, The First Affiliated Hospital, Nanchang University, Nanchang, Jiangxi, China; ^3^Department of Neurosurgery, Xuan Wu Hospital, Capital Medical University, Beijing, China; ^4^Department of Neurology, PLA General Hospital, Beijing, China

**Keywords:** intracranial aneurysms, dissection, vertebrobasilar artery, endovascular treatment, recurrence

## Abstract

**Background:**

We aimed to evaluate the risk factors of recurrence after stent(s)-assisted coiling (SAC) of intracranial vertebrobasilar dissecting aneurysms (VBDAs) based on 168 consecutive patients.

**Methods:**

Between January 2011 and December 2015, 168 consecutive patients with 170 intracranial VBDAs, which were treated by SAC, were recruited from four high-volume centers. We used multivariate logistic regression to examine factors that affected recurrence of VBDAs.

**Results:**

The mean duration of clinical follow-up of the 168 patients was 7.81 months (range, 3–24 months). Of the 168 patients, 4 (2.38%) suffered from intraoperative complications and 16 (9.52%) had postoperative complications. Two (1.19%) had severe disability. Imaging follow-up was available for 168 patients (170 VBDAs), with a mean duration 7.81 months, and 24 (14.12%) cases of recurrence of aneurysms were noted. Aneurysm size and metal coverage of stent(s) at the neck were independent predictors of recurrence after SAC by logistic regression analysis.

**Conclusion:**

This multicenter cohort study shows that aneurysm size and the metal coverage of stent(s) at aneurysmal neck are independent factors associated with recurrence of VBDAs after SAC.

## Introduction

Vertebrobasilar dissecting aneurysms (VBDAs) are uncommon lesions of which treatment is challenging and represent 3.3% of all intracranial aneurysms (1). VBDAs can cause subarachnoid hemorrhage (SAH) and posterior circulation ischemia with high morbidity and mortality (2–5). Endovascular treatment, including deconstructive and reconstructive treatment (stent-assisted coiling [SAC] or stent only), has emerged as a major therapeutic option for VBDAs (6). However, with the increasing number of cases being treated, recurrence of VBDAs after SAC is gradually being found (2, 7–9). Identifying risk factors of VBDA recurrence after SAC is an important issue and would be helpful for endovascular treatment. The Raymond Scale, the VBDA size, and the branch artery involved are risk factors of VBDA recurrence after SAC (10–14). However, most authors of previous studies on VBDAs have made their conclusion based on single-center data and limited cases. This study aimed to identify risk factors of recurrence after SAC in a large multicenter sample of patients with VBDAs. In this retrospective study, we examined baseline information, the details of endovascular treatment, and the clinical outcomes in 168 consecutive patients with VBDAs at four high-volume medical centers.

## Materials and Methods

The retrospective study was approved by the Institutional Review Board and carried out in four research units. All included patients or their relatives provided written informed consents during hospitalization and the privacy of the patients were strictly protected.

### Selection of Patients and the Population

Between January 2011 and December 2015, endovascularly treated dissecting aneurysms at four high-volume centers were included in an online retrospective multicenter database. The medical records and image data of each patient were reviewed. The VBDAs were diagnosed by digital subtraction angiography (DSA), computed tomographic angiography, magnetic resonance angiography, and magnetic resonance imaging were used to diagnose the VBDAs. Ultimately, 168 consecutive patients with 170 intracranial VBDAs, which were treated by SAC, were included in this study. The exclusion criteria included the following: (1) a history of traumatic and iatrogenic injury; (2) a history of deconstructive treatment; (3) a history of reconstructive treatment with sole stent(s); (4) untreated VBDAs; and (5) loss to any clinical follow-up. The following factors were analyzed: age, sex, cigarette smoking, alcohol intake, hypertension, diabetes mellitus, aneurysm size, complications, immediate angiography results, follow-up interval, and angiographic and clinical follow-up results (Table 1).

**Table 1:** Baseline characteristics of the 168 patients with VBDAs.

Types	Total
No. (%)	168 (100)
Age	51.92 ± 11.01
Men (%)	133 (79.17)
**Presentation**	
SAH (%)	47 (27.98)
TIA/stroke ± headache (%)	33 (19.64)
Compressive ± headache (%)	21 (12.50)
Incidental (%)	67 (39.88)
**mRS (pre-operation)**	
0–1	149
2–4	18
5–6	1
**mRS (follow-up)**	
0–1	157
2–4	9
5–6	2
Follow-up period (months)	7.81 ± 4.30

*SAH, subarachnoid hemorrhage; TIA, transient ischemic attacks; mRS, modified Rankin Scale; VBDAs, vertebrobasilar dissecting aneurysms*.

### Endovascular Procedures

Endovascular procedures were performed under general anesthesia. For patients with acutely ruptured VBDAs, antiplatelet medication (300 mg aspirin and 300 mg clopidogrel) was preloaded orally or through a stomach tube 2 h before the procedure. For patients with unruptured VBDAs, dual antiplatelet (75 mg of clopidogrel and 100 mg of aspirin) therapy was administered each day for 3 days. All of the patients received systemic intravenous heparin after the guiding catheter was placed. Self-expanding neurovascular stents were used for reconstruction of the entire dissected segment and coils were used to embolize the aneurysm. Dual antiplatelet agents (75 mg clopidogrel and 100 mg aspirin) were provided orally once daily for 6 weeks after the endovascular procedure. Following this therapy, 100 mg aspirin was continued for the next 6 months.

### Materials

Various types of detachable coils, such as the Matrix coil (Cordis, New Brunswick, NJ, USA) and Microplex coil (MicroVention, Aliso Viejo, CA, USA), were used in endovascular treatment. Self-expanding neurovascular stents, such as Enterprise (Cordis Neurovascular, Miami, FL, USA), Solitaire AB (Ev3, Irvine, CA, USA), and Low-profile Visualized Intraluminal Support (MicroVention Terumo, Tustin, CA, USA) stents, were used to reconstruct the dissected artery. Metal coverage of the stents was estimated by stent type and diameter of the parent artery (15, 16). For multiple stents, the metal coverage was acquired by adding the metal coverage of deployed stents together.

### Angiography Assessment

Digital subtraction angiography findings were reviewed by two experienced interventional neuroradiologists. Endovascular treatment details, including immediate obliteration grade, type of stents used, and important branches involved, were recorded, as were angiographic follow-up outcomes. Angiographic results were evaluated with the Raymond grading system as follows: grade 1, complete occlusion; grade 2, residual neck; and grade 3, residual aneurysm (17). The important vessel branches were the posterior inferior cerebellar artery, anterior inferior cerebellar artery, superior cerebellar artery, posterior cerebral artery, anterior spinal artery, or other large perforating arteries.

### Follow-up

Angiographic follow-up was performed with DSA at approximately 6 months. The patient was then with an MRA or CTA followed up annually. Angiographic recurrence means a substantial increase in the contrast medium-filled portion of the dissecting aneurysm compared with the control angiogram just after the initial treatment. The patient clinical outcome was measured by the modified Rankin Scale (mRS) score at follow-up visits or by a telephone interview.

### Statistical Analysis

Statistical analysis was performed using IBM SPSS statistics version 22.0. The one-sample Kolmogorov–Smirnov test was used to test the normality of the data distribution for quantitative data. Data are expressed as the mean ± SD or median (quartile). The groups were compared using the independent sample *t*-test, Mann–Whitney *U* test, or the χ^2^ test appropriately. Univariate analysis was performed to determine the association of angiographic or clinical results with other factors. Then, variables with *P* < 0.20 in univariate analyses (initial Raymond grade, aneurysm size, and the metal coverage of stent(s) at aneurysm neck) were candidates for inclusion in multivariate logistic regression analysis. *P* < 0.05 was considered statistically significant.

## Results

A total of 168 patients (52 women and 176 men) with 170 VBDAs were enrolled in this study. Two patients had two aneurysms. The mean age of the patients was 51.92 ± 11.01 years.

### Endovascular Treatment Complications

Of the 168 patients (170 aneurysms), 4 (2.38%) suffered from intraoperative complications, and 16 (9.52%) had postoperative complications (Table 2). The four intraoperative complications included stent thrombosis in one, aneurysm rupture in one, serious vertebral artery vasospasm in one, and iatrogenic dissection of the vertebral artery in one patient. The 16 postoperative complications included symptomatic cerebral infarction in seven, pneumonia in three, rupture of the VBDAs after endovascular treatment in two, asymptomatic stent thrombosis in two, and hydrocephalus in two patients.

**Table 2:** Perioperative complications of the 168 patients with 170 vertebrobasilar dissecting aneurysms.

Treatment	Stent(s)-assisted coiling
Aneurysm no.	170
Intraoperative complications	4
In stent thrombosis	1
Intraoperative hemorrhage	1
Stent drifting	1
Cerebral vasospasm	1
Postoperative complications	16
Hemorrhagic	2
Ischemic	7
Compression	2
Others	5

### Results of Clinical Follow-up

168 patients participated in the clinical follow-up (mean follow-up period: 7.81 ± 4.30 months) (Table 1). At the last follow-up, 157 patients had an mRS of 0 or 1, nine patients had an mRS of 2 to 4, and two patients had an mRS of 5 or 6. Two (1.19%) patients had severe disability.

### Results of Radiographic Follow-up and Risk Factors for Aneurysm Recurrence

**Figure 1:**
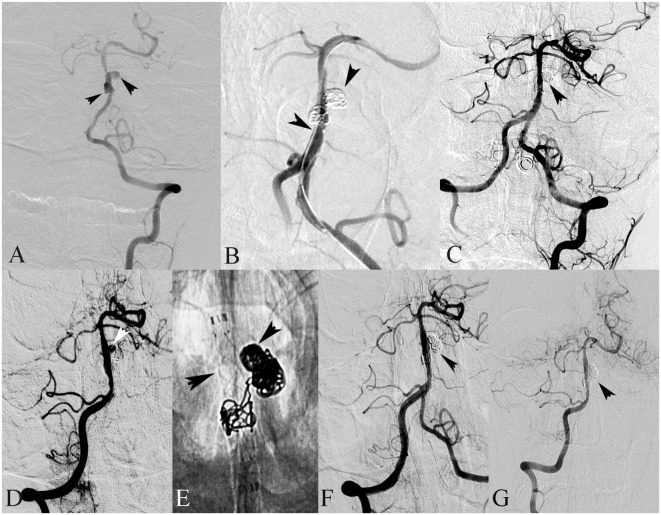
A 18-year-old male presented with hemiplegia and alalia. **(A)** Left vertebral angiography in frontal view demonstrates dissection of the basilar artery. **(B)** The basilar dissecting aneurysm was treated with stent(s)-assisted coiling (SAC) (double Enterprise stents). **(C)** Frontal view of the left vertebral artery showed complete occlusion after SAC. **(D)** Follow-up angiography after 3 months showed recanalization of the dissecting aneurysm. **(E)** Then, the dissecting aneurysm was retreated with SAC (LVIS stent). **(F)** Frontal view of the right vertebral artery showed complete occlusion after SAC. **(G)** Follow-up angiography after 6 months showed complete occlusion of the dissecting aneurysm.

**Figure 2:**
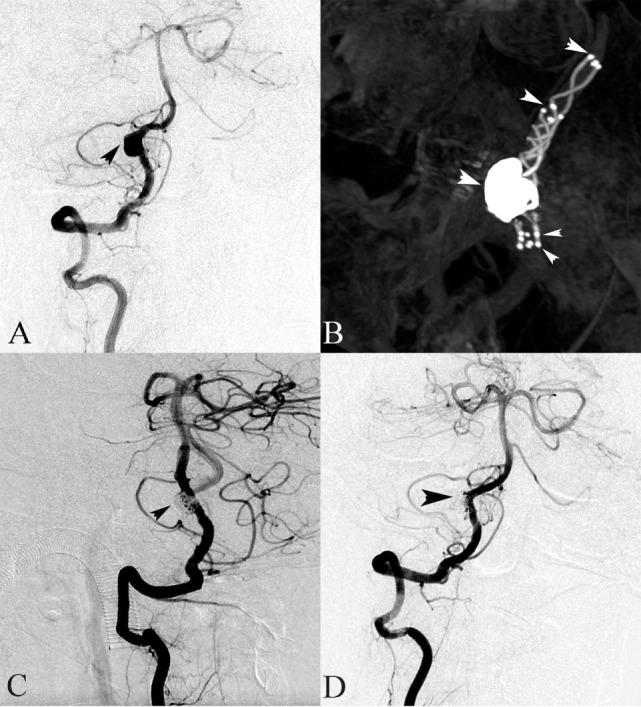
A 64-year-old female presented with headache. **(A)** Right vertebral angiography showed a dissecting aneurysm. **(B)** Stent(s)-assisted coiling (SAC) (double LVIS stents) were performed. **(C)** The right vertebral angiography after SAC treatment demonstrated complete occlusion. **(D)** Follow-up digital subtraction angiography after 6 months showed a complete occlusion of the dissecting aneurysm sac.

Angiographic follow-up was available for all 168 patients (170 VBDAs), with a mean duration of 7.81 months (range, 3–24 months), and 24 (14.12%) aneurysm recurrences were observed. There were 146 VBDAs with residual neck or residual aneurysm (Raymond scale grade 2 or 3) after SAC treatment. According to the follow-up DSA images, the residual neck or residual aneurysm improved or kept stable in 124 VBDAs and worsen in 22 VBDAs. The recurrence rate and the risk factors based on the 170 aneurysms are shown in Table 3. Univariate analysis showed that initial Raymond grade, aneurysm size, and metal coverage of the stent(s) at the aneurysm neck were related to the recurrence rate. Larger aneurysm size [odds ratio (OR) = 1.098; 95% confidence interval (CI), 1.034–1.166; *P* = 0.002] and the lower metal coverage of stent(s) at neck (OR = 0.914; 95% CI, 0.850–0.982; *P* = 0.014) were independent predictors of recurrence in the logistic regression analysis (Figures 1 and 2).

**Table 3:** Risk factors for angiographic recurrence after endovascular reconstructive treatment in IDAs (*n* = 170).

Variables	Improved or stable (*n* = 146)	Recurrent (*n* = 24)	*P* value	OR, 95% CI	*P* value for logistic regression
Age (years)	52.24 ± 10.94	50.00 ± 11.96	0.360		
Gender			0.679		
Male	115	18			
Female	31	6			
Risk factors^a^			0.549		
<2	106	16			
≥2	40	8			
Aneurysm size (mm)^b^	8.2 (12.4)	11.4 (15.3)	0.022	1.098 (1.034, 1.166)	0.002
Rupture			0.411		
Yes	37	8			
No	109	16			
Raymond scale			0.047		0.069
1	22	2			
2	54	5			
3	70	17			
Important branch artery involved			0.348		
Yes	41	9			
No	105	15			
Metal coverage at neck (%)^b^	17.84 (23.00)	11.89 (17.84)	0.017	0.914 (0.850,0.982)	0.014

*OR, odds ratio; CI, confidence interval*.

*^a^Risk factors include high blood pressure, smoking, diabetes mellitus, and high cholesterol level*.

*^b^The data were expressed as median (quartile). Mann–Whitney *U* test was used*.

## Discussion

The endovascular method has become the primary treatment for intracranial VBDAs (1, 18). However, the relatively high recurrence rate of VBDAs is a major problem after reconstructive treatment (19). In previous studies, posterior circulation dissecting aneurysms have a higher recanalization rate than anterior circulation dissecting aneurysms (33 versus 6%) and intracranial dissecting aneurysms recur at a higher rate (18%) than saccular aneurysms (8.3–14%) (19–23). This may be because dissection in posterior was prone to form aneurysm and dissecting aneurysm have more complicated anatomic configurations, such as pearl-and-string configurations, intramural hematomas, and intimal flap which increase the difficulty of treatment (22–24). There have been many small case series of treating VBDAs with endovascular methods, but few series have mainly focused on recurrence of VBDAs after reconstructive treatment (25, 26). This is because the morbidity of dissecting aneurysms is lower than that of saccular aneurysms (1). Additionally, the internal structure of dissecting aneurysms is more complicated than that of a saccular aneurysm, making computer modeling and computer fluid hemodynamic analysis more difficult. In this study, we examined 168 patients with 170 VBDAs who were treated by SAC at four high-volume medical centers, with the aim of identifying risk factors of recurrence of VBDAs after SAC. We found that larger aneurysm size and the lower metal coverage of stent(s) were associated with recurrence of VBDAs after SAC. To our knowledge, the present case series is the largest series of VBDAs with angiographic follow-up treated by SAC.

Aneurysm size was an independent predictor of progression of aneurysms in our study, with larger aneurysms associated with a higher progression rate. This finding has been reported by many authors (11–13). Large VBDAs, which are thought to develop from small, unstable VBDAs, might be even less stable than small VBDAs. Additionally, large VBDAs generally have a longer dissecting segment and aneurysm neck, making them difficult to completely occlude. These findings might explain the association between larger aneurysms and higher progression rates after reconstructive endovascular treatment.

The obliteration grade of the aneurysm is thought to be an important factor for a low rate of occurrence of aneurysms after reconstructive endovascular treatment (13). We always strive to achieve high coil packing density of the aneurysm in our clinical practice. The limited number of cases from a single institute might explain why this factor was excluded from the risk factors for progression of aneurysms in multivariate logistic analysis.

Metal coverage of the stent(s) at the neck was also an important predictor of VBDA recurrence in our study, with a higher metal coverage rate associated with a lower recurrence rate. This finding is consistent with many studies, which showed that an increase in the stent metal coverage rate or stent quantity can reduce the aneurysm recurrence rate. In Zhao et al. study, overlapping stents which has a higher metal coverage of stents was associated with lower recurrent rate than single stent (19). An increase in metal coverage of stent(s) at the neck can reduce the recurrence rate because of the following reasons. An increase in metal coverage of stent(s) at the neck can reduce the blood impact, which can prevent coil mass compaction. Additionally, an increase in metal coverage of stent(s) at the neck can decrease intra-aneurismal wall shear stress, which prevents aneurismal growth (27, 28). Inspiration can be acquired from that higher metal coverage of stent(s) at neck could reduce the VBDAs recurrence rate. For a lesion that is difficult to completely occlude, an increase in metal coverage of the stent(s) at the neck (overlapping stents or a stent with lower porosity) may be an alternative for preventing recurrence of VBDAs. This viewpoint has been well documented in flow diverter devices (29). However, a flow diverter stent was not used in this study because the China Food and Drug Administration have not yet approved its application in the posterior cerebral circulation. Additionally, we have only had initial experience of application of this stent in the anterior circulation.

There are some limitations to this series, such as limited number of cases, the retrospective design, different interventional materials used, and patient selection bias. Moreover, it is very difficult to get exact values of metal coverage in clinical practice.

## Conclusion

This multicenter cohort study indicated that aneurysm size and the metal coverage of stent(s) at aneurysmal neck were independent factors associated with recurrence of VBDAs after SAC.

## Ethics Statement

The ethics committee of our institutes approved this study and informed consent was obtained from each study patient or their relatives.

## Author Contributions

KW and ZT contributed to the preparation of the manuscript and data collection. JC, JL, YW, HZ, and JW contributed to data analysis and interpretation. YZ and XY contributed to the experimental design and manuscript revision.

## Conflict of Interest Statement

The authors declare that the research was conducted in the absence of any commercial or financial relationships that could be construed as a potential conflict of interest.
